# Balancing Innovation and Neophobia in the Production of Food for Plant-Based Diets

**DOI:** 10.3390/foods11121702

**Published:** 2022-06-10

**Authors:** Antonella Pasqualone

**Affiliations:** 1 Food Science and Technology Unit, Department of Soil, Plant and Food Science (DISSPA), University of Bari ‘Aldo Moro’, Via Amendola 165/a, 70126 Bari, Italy; antonella.pasqualone@uniba.it; 2 Brussels Institute of Advanced Studies (BrIAS) Fellow 2021/22, 1050 Elsene, Brussels, Belgium

In addition to vegetarians and vegans, plant-based diets are adopted by flexitarians or semi-vegetarians, i.e., people who choose to substitute animal products with vegetable options for ethical, religious and health reasons, without permanent restriction of animal foods. Alcorta et al. [1] attracted the interest of international researchers by highlighting the challenges and innovations related to the preparation of foods for plant-based diets. The significance of their critical analysis is underlined in this editorial.

The number of consumers who are reducing the intake of animal-based foods is increasing globally, resulting in a growing market for plant-based products. In addition to including fruits, vegetables, grains and legumes, plant-based diets include a wide range of foods that mimic the sensory characteristics of conventional animal products (meat, milk and dairy products, and egg), but are generally made from non-animal protein sources [1].

Proteins from pulses, wheat gluten and mycoproteins are the most common ingredients in the preparation of meat analogs. Pulses-based protein isolates or concentrates are obtained by wet or dry fractionating pulse flours, the dry process being more eco-sustainable because does not consume water [2]. Their texturization is then carried out by extrusion cooking [3]. Seitan, or wheat meat, is prepared from gluten, which has cohesive and chewy features similar to meat. In mycoproteins, the meat-like texture is imparted by their filamentous structure [4]. In addition to preparing meat analogs from plant-based sources, a different approach involves producing “regular” meat but with minimal use of animals using a meat culture. This technique makes it possible to produce muscle tissue by growing animal cells in a culture medium [5]. While this approach may be unacceptable to vegans and vegetarians, it could be acceptable for flexitarians. Other innovations with very promising futures involve the use of edible insects or microalgae. Insects are rich in proteins, polyunsaturated fatty acids (PUFAs) and antioxidants, and have the potential to be used as meat substitutes or dietary supplements [6]. Additionally, microalgae (*Chlorella* sp., *Arthrospira* sp. and *Schizochytrium* sp.) are very rich of high biological value proteins (up to 70%) and PUFAs, and can be effectively used as dietary supplements or protein ingredients [7].

Plant-based milk alternatives are water solutions or fine suspensions with a similar appearance to cow’s milk, prepared from various grains by grinding them, soaking, filtering and finally homogenizing the water extract [8]. In the manufacture of cheese alternatives, plant proteins and vegetable oils are commonly used. Legume proteins have been explored, but they need to be fermented to reduce the content of anti-nutritional factors and the typical bean-like flavor [9]. Egg alternatives include mixtures of gums and proteins able to mimic the emulsifying and foaming properties of egg to be used as ingredients in other food products such as mayonnaise [10].

Though there is an increasing demand for plant-based foods, consumers may be suspicious and reject novel or unfamiliar foods. This phenomenon is called “food neophobia” [11]. Alcorta et al. [1] highlight that “food neophobia can be partially alleviated through informative and clear labeling”. Compared to plant-based meat alternatives, cultured meat is often perceived as unnatural and less acceptable [12]. Insects, at least in Western countries, are even considered disgusting. In that case, product perception could be improved by specifying the exact protein source in the ingredients list and accompanying the food products with information explaining the environmental advantages of the innovation, as well as emphasizing the specific qualities of the final product. Additionally, high levels of processing, such as those required to improve texture, or the presence of preservatives can trigger food neophobia.

After overcoming the neophobia, however, food products must fundamentally be pleasant to taste, otherwise consumers are not likely purchase them. One of the main challenges of plant-based meat analogs is to reproduce the sensory properties of meat, particularly regarding taste; this may be less important for vegans and vegetarians, but it is fundamental to flexitarians [13]. Regarding texture, consumers find that processed meat analogs, such as plant-based burgers and sausages, are more acceptable than unprocessed meat substitutes, such as vegetable steak [13], because processed products have a similar texture whether they are of animal or plant origin. For milk alternatives, taste and sugar content are the main factors influencing acceptability [14].

However, consumer acceptability is not the only challenge that foods for plant-based diets must overcome. Other issues include the possible deficiency of some nutrients such as B12 and D vitamins, iron, calcium, and high-biological-value proteins. These aspects must be carefully considered both in the formulation of food products, adopting adequate measures for micronutrient fortification and amino acid complementation, and by nutritionists, in the context of an overall well-balanced daily dietary intake.

Furthermore, although plant-based food products are generally more environmentally friendly than their animal-based counterparts, the sustainability gains from these products are affected by the degree of processing, as highly processed foods have a greater environmental impact than less processed ones due to the higher energy consumption. The reuse and upcycling of plant by-products and waste, often rich in bioactive compounds [15], can enhance the sustainability of these foods. Functional plant-based foods and beverages are an emerging segment of the food market.

In conclusion, despite the growing consumer interest in plant-based diets, the processing technology of alternative foods still needs to be optimized, with the goal of improving their sensory features—the main obstacle to acceptability. Furthermore, comprehensive information campaigns are needed to reduce the neophobia of the most innovative food products, such as cultured cells, insects and microalgae, changing eating habits. The increase in the world population, together with the depletion of natural resources, global warming, and conflicts, determine the urgency of the transition to a more sustainable food system and a reduction in food loss and waste. We are all involved—producers and consumers.
